# Linking adults and cystacanths of a new species of *Rhadinorhynchus* Lühe, 1911 (Acanthocephala: Rhadinorhynchidae) from the Pacific coast of Mexico by using morphological and molecular data

**DOI:** 10.1007/s11230-024-10205-9

**Published:** 2024-12-05

**Authors:** Mayra I. Grano-Maldonado, Ana L. Sereno-Uribe, José Carlos Hernández Payán, Gerardo Pérez-Ponce de León, Martín García-Varela

**Affiliations:** 1 Facultad de Ciencias del Mar, Universidad Autónoma de Sinaloa, Av. Claussen s-n, Mazatlán, Sinaloa México; 2 Departamento de Zoología, Instituto de Biología, Universidad Nacional Autónoma de México, Ciudad Universitaria, C.P. 04510 Ciudad de México, México; 3 Posgrado en Ciencias del Mar y Limnología, Universidad Nacional Autónoma de México, Av. Ciudad Universitaria 3000, C.P. 04510 Coyoacán, Ciudad de México, México; 4 Escuela Nacional de Estudios Superiores unidad Mérida, Universidad Nacional Autónoma de México, Tablaje Catastral N°6998, Carretera Mérida-Tetiz Km. 4.5, Municipio de Ucú, 97357 Mérida, Yucatán, México

## Abstract

During parasitological surveys of marine fishes and zooplankton in localities of the Northwestern Pacific coast of Mexico, 28 Gafftopsail pompano (*Trachinotus rhodopus* Gill) and 50 mysid crustaceans (*Metamysidopsis frankfiersi* Hendrickx & Hernández-Payán) we identified to be infected with adults and cystacanths, respectively of an acanthocephalan morphologically corresponding to the genus *Rhadinorhynchus* Lühe, 1911. DNA sequences of the small (SSU) and large (LSU) subunits of ribosomal DNA, and cytochrome c oxidase subunit 1 (*cox 1*) from mitochondrial DNA were obtained. Phylogenetic analyses revealed that the newly sequenced individuals in a clade with *Rhadinorhynchus* sp. from carangids in other localities of the Pacific coast of Mexico; together, all these individuals formed an independent lineage that is recognized herein as a new species, *Rhadinorhynchus trachinoti* **n. sp.** The new species is morphologically distinguished from the other 38 congeners by having a long and cylindrical proboscis armed with 12 longitudinal rows bearing 16–18 hooks each. The ecological information gathered from the parasites, together with genetic evidence, confirms that the Gafftopsail pompano is the definitive host of *R. trachinoti* **n. sp.**, while mysid crustaceans serve as the intermediate host. Current records also indicate that *R. trachinoti* **n. sp.** is distributed along the Pacific coast of Mexico, from Mazatlán, Sinaloa in the north to Puerto Angel, Oaxaca in the south. This distribution aligns with the Mexican Coastal Current, which extends from the Gulf of Tehuantepec in Oaxaca to the entrance of the Gulf of California and southern Baja California.

## Introduction

The helminth parasite diversity of marine fishes along the Pacific coast of Mexico has been documented for several decades, revealing a large biodiversity for some helminth groups such as monogeneans, digeneas, cestodes, nematodes and, to a lesser extent, acanthocephalans (Pérez-Ponce de León et al., 2007; García-Prieto et al., 2010; Violante-González et al., 2016, 2020; Villalba-Vasquez et al., 2022; Martínez-Flores et al., 2023). The first record of an acanthocephalan parasitizing marine fishes is Mexico dates back to 1940 when the species *Filisoma bucerium* Van Cleave 1940 was reported from the Cortez Sea chub (*Kyphosus elegans*) (Peters) in Socorro Island (Van Cleave, 1940); since then, other seven adult acanthocephalans have been recorded, including *Echinorhynchus gadi* Zoega in Müller, 1776; *Koronacantha mexicana* Monks & Perez-Ponce de León, 1996; *K. pectinaria* (Van Cleave, 1940); *Floridosentis pacifica* Bravo-Hollis, 1969; *Neoechinorhynchus roseum* Salgado-Maldonado, 1978; *Pomphorhynchus rocci* Cordonnier & Ward, 1967, and an unidentified species of the genus *Rhadinorhynchus* Lühe, 1911; these species represent 12.3% of the biodiversity of acanthocephalans in Mexico (see García-Prieto et al., 2010; Martínez-Flores et al., 2023; Osuna-Cabanillas et al., 2024).

Members of the genus *Rhadinorhynchus* are endoparasites of marine fishes throughout the world (Amin et al., 2011; Amin, 2020). Morphologically, the genus is characterized by having a long and cylindrical proboscis, with hooks of different sizes, trunk cylindrical, spinose anteriorly in one or two fields, neck prominent, proboscis receptacle longer than proboscis, lemnisci digitiform, gonopore terminal or subterminal; males possessing four cement glands and females possessing eggs with polar prolongation. The genus currently contains 39 species (Amin et al., 2011, 2019; Lisitsyna et al., 2019; Amin, 2020) although the WoRMS database consider 49 species (Gibson and Wayland, 2024) indicating that the species composition within the genus remains unsettled. It is well known that species of *Rhadinorhynchus* have diversified in marine environments and that they can infect fishes from the families Carangidae, Salmonidae, Scombridae Terapontidae, Tetradontidae, and Trichuridae (Amin et al., 2019; Amin, 2020; Violante-González et al., 2020; Santos-Bustos et al., 2021; Osuna-Cabanillas et al., 2024).

Carangids (jacks and pompanos) play a principal role in the marine ecosystem and are economically important fish. Particularly, the Gafftopsail pompano (*Trachionotus rhodopus* Gill) is distributed mostly in marine tropical and subtropical waters across the Americas; it feeds mainly on crustaceans (zoea larvae, copepods, isopods, mysids, amphipods, and decapods) fishes, annelids, mollusks, sipunculids and algae (Thompson et al., 1979; Danemann, 1993). Due to its high abundance and common landings of artisanal fishing fleets, it has been targeted for metazoan parasites studies in some localities of Pacific coast of Mexico (e.g., Martínez-Flores et al., 2023; Osuna-Cabanillas et al., 2024).

During a survey of parasitic helminths on the North Pacific coast of Mexico, adult specimens of an acanthocephalan were recovered from the digestive tract of Gafftopsail pompano (*T. rhodopus*), and cystacanths were obtained from the hemocoel of a recently described species of mysid crustacean, *Metamysidopsis frankfiersi* (Hendrickx & Hernández-Payán, 2023). After a morphological examination of worms from both developmental stages, adults and cystacanths were identified as an undescribed species of *Rhadinorhynchus* sp. Therefore, the objectives of this study were: *i*) to describe a new species of *Rhadinorhynchus* from the Gafftopsail pompano (*T. rhodopus*) from the North Pacific coast of Mexico; *ii*) to link the cystacanths recovered from the mysid (*M. frankfiersi*) with the adult specimens from fish; *iii*) to test the systematic position of the new species among other congeners by using small (SSU) and large (LSU) subunit from nuclear ribosomal DNA and cytochrome c oxidase subunit 1 (*cox1*) from mitochondrial DNA; and *v*) to discuss the ecological features of the infection of the new species in their definitive and intermediate hosts.

## Materials and methods

*Sample collection*

During several field expeditions between 2021 and 2022, a total of 87 fish (27.55 ± 4.67 cm length) belonging to Gafftopsail pompano (*T. rhodopus*) were collected off Mazatlán Bay in the southeastern Gulf of California (23° 12′ 37.15″ N, 106° 25′ 28.92′ W) with the help of local fisherman. In May 2023 a total of 1,613 mysids (*M. frankfiersi*) were collected in Playa Novillero, Nayarit, Mexico (22° 22′ 41.3″ N, 105° 41′ 11.4″ W) in the wave or breaking zone at approximately 1 m depth, with a small zooplankton net (0.5 mm mesh opening). The definitive hosts were dissected, and their viscera were placed in separate Petri dishes with a 0.85% saline solution and examined under a dissecting microscope. The adult acanthocephalans were removed from the intestine, washed in a 0.85% saline solution, placed in distilled water at 4°C overnight and subsequently fixed and preserved in 70% ethanol. The intermediate hosts were collected and preserved in containers with 70% ethanol; in the laboratory, mysids were observed under the stereomicroscope, and cystacanths were removed from the hemocoel and placed in 96% ethanol.

*Morphological analyses*

Adult and cystacanths were gently punctured with a fine needle, stained with Mayer’s paracarmine, destained in 70% acid ethanol, dehydrated in a graded ethanol series, cleared in methyl salicylate, and mounted on permanent slides with Canada balsam. Acanthocephalans were analyzed with a Leica DM 1000 LED microscope equipped with a bright field (Leica, Wetzlar, Germany). Acanthocephalans were initially identified by conventional morphological criteria following the key of Amin et al. (2011). For scanning electron microscopy (SEM), two adults and a single cystacanth were individually dehydrated with an ethanol series, critical point dried, sputter coated with gold, and examined with a Hitachi Stereoscan Model S-2469N scanning electron microscope operating at 15 kV at the Instituto de Biología, Universidad Nacional Autónoma de México (UNAM).

*DNA sequence generation*

**Figure 1:**
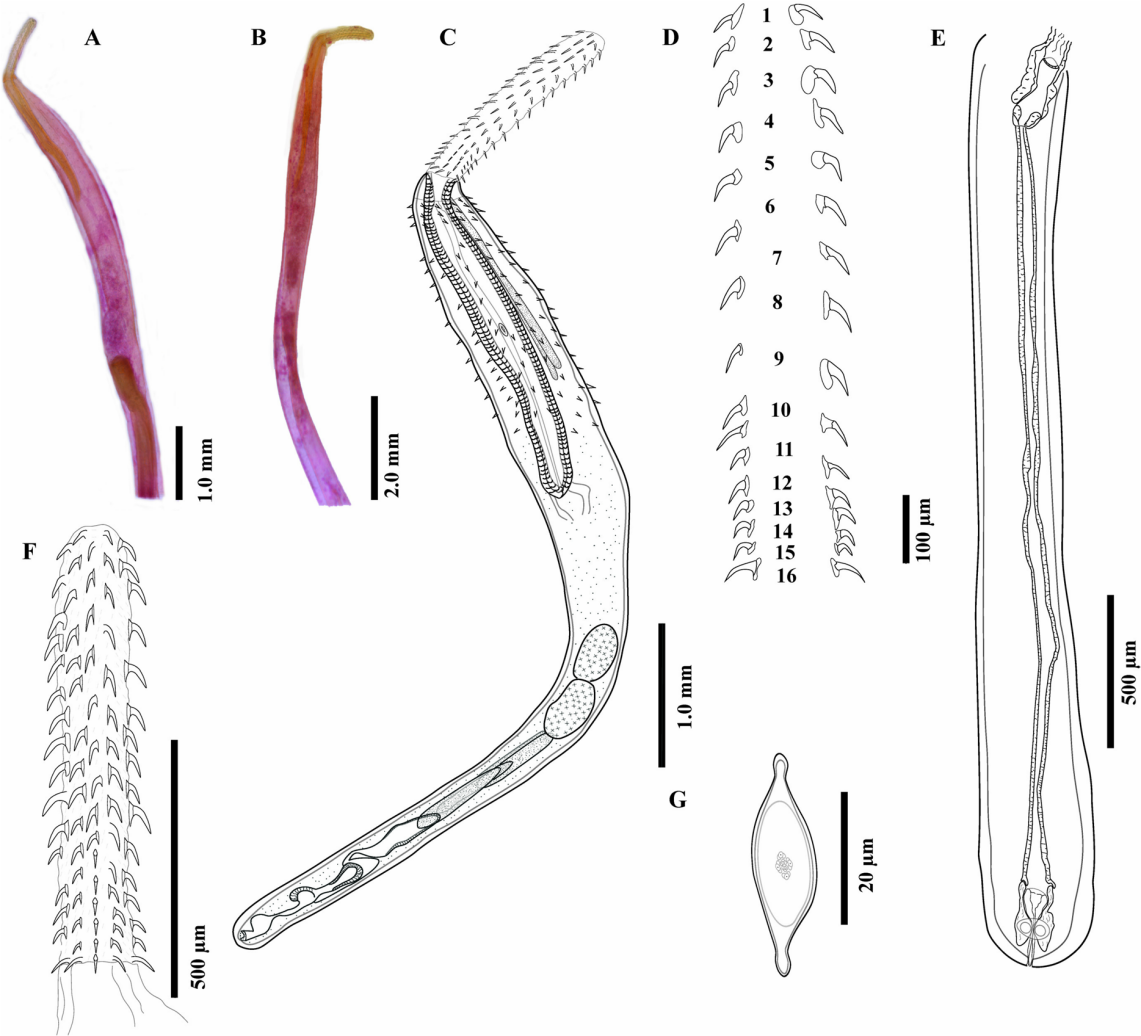
Hologenophore, adult male (**A**); female, (**B**) of ***Rhadinorhynchus trachinoti*** **n. sp.**, from *Trachinotus rhodopus* from off Mazatlán, Sinaloa, Mexico. Drawings of whole adult male, whole worm (**C**); Proboscis hooks (**D**); Female reproductive system (**E**); Proboscis (**F**); Egg (**G**)

**Figure 2:**
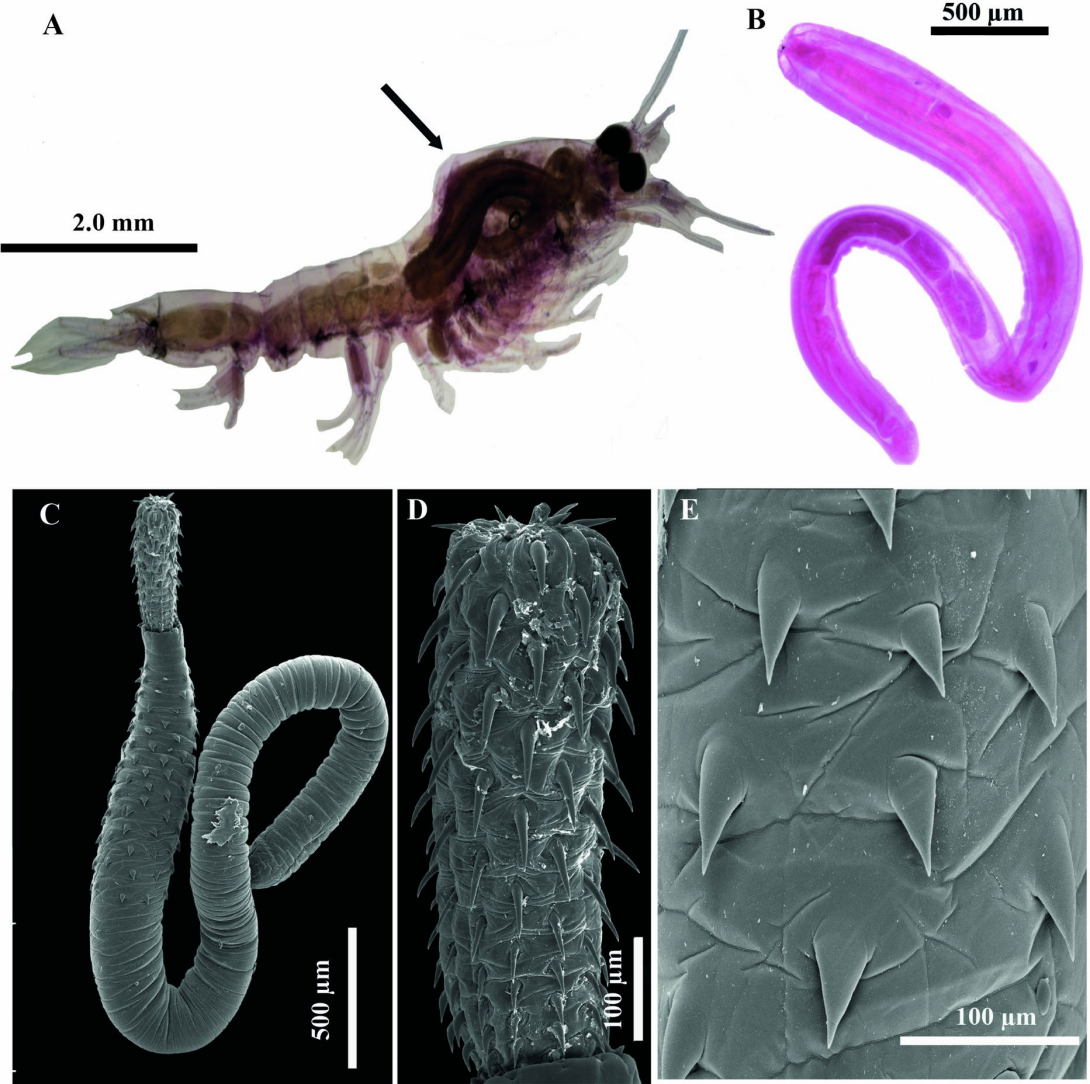
Photographs of the mysid *Metamysidopsis frankfiersi* harboring the cystacanth of ***Rhadinorhynchus trachinoti*** **n. sp.** (**A**); Photograph of a mounted stained male cystacanth (**B**); Scanning electron micrographs of the cystacanth whole worm (**C**); Proboscis (**D**); Tegumental spines from anterior region of the trunk of the cystacanth (**E**). Arrow indicates the cystacanth

Before DNA extraction, a tissue fragment was cut from two adult acanthocephalans whereas the rest of the body was stained with Mayer’s paracarmine and mounted on permanent slides with Canada balsam (*hologenophore*, *sensu* Pleijel et al., 2008) (Fig. 1A–B). Before DNA extraction, two infected mysids (*M. frankfiersi*) with cystacanths were mounted on a microscope slide, and images were taken as references with a bright field Leica DM 1000 LED microscope (Leica, Wetzlar, Germany) (Fig. 2A). After the cystacanths were removed from the hemocoel each image was linked with its genomic DNA, (*photogenophore sensu* Andrade-Gómez & García-Varela, 2021). Genomic DNA was isolated, following the protocol described by García-Varela et al. (2024). Two regions of nuclear ribosomal DNA (rDNA) and one mitochondrial DNA region (mtDNA) were amplified using the polymerase chain reaction (PCR). A near complete fragment from the small subunit from 18S rDNA (~1,800 bp; SSU) was amplified using two overlapping PCR fragments of 1,000 bp: the SSU amplicon 1 using the forward primer 5′-AGA TTA AGC CAT GCA TGC GT-3′ and reverse primer5′-AAC TTT TCG TTC TTG ATT AA TG-3′ and, the SSU amplicon 2 using the forward primer 5′-GCA GCG CGG TAA TTC CAG CTC-3′ and reverse primer 5′-GCA GGT TCA CCT ACG GA AA-3′ (García-Varela & Nadler, 2005). A near complete fragment of the large subunit from 28S rDNA (~2,900 bp; LSU) was amplified using three overlapping PCR fragments of 1200-1300 bp: the LSU amplicon 1 using the forward primer 5′-CAA GTA CCG TGA GGG AAA GTT GC-3′ and reverse primer 5′-CAG CTA TCC TGA GGG AA AC-3′, the LSU amplicon 2 using the forward primer 5′-ACC CGA AAG ATG GTG AAC TA TG-3′ and the reverse primer 5′- CTT CTC CAA CGT CAG TCT TC AA-3′, and, the LSU amplicon 3 using the forward primer 5′- CTA AGG AGT GTG TAA CAA CTC ACC-3′ and reverse primer 5′-CTT CGC AAT GAT AGG AAG AG CC-3′ (García-Varela & Nadler, 2005). Finally, the cytochrome c oxidase subunit 1 (*cox 1*) from the mitochondrial DNA was amplified using the forward primer 5′-AGTTCTAATCATAA(R)GATAT(Y)GG-3′ and reverse primer 5′ -TAAACTTCAGGGTGACCAAAAAATCA-3′ (Folmer et al., 1994). PCR amplifications were performed in a total volume of 25 μl containing 2 μl of each primer, 10 pmol/ μl, 2.5 µl of 10X buffer, 1.5 μl of 2 mM MgCl_2_, 2 μl of the genomic DNA, and 1U of Taq DNA polymerase (Platinum Taq, Invitrogen Corporation, California, United States). PCR cycling parameters for rDNA amplification included denaturation at 94 °C for 3 min, followed by 35 cycles of 94 °C for 1 min, annealing at 50–58 °C (optimized for each fragment amplified) for 1 min, and extension at 72 °C for 1 min, followed by a post-amplification incubation at 72 °C for 7 min. Sequencing reactions were performed with the primers mentioned above using ABI Big Dye (Applied Biosystems, Boston, Massachusetts) terminator sequencing chemistry. Reaction products were separated and detected using an ABI 3730 capillary DNA sequencer. Contigs were assembled and base-calling differences were resolved using Codoncode Aligner version 9.0.1 (Codoncode Corporation, Dedham, Massachusetts).

*Phylogenetic analyses*

Newly generated sequences of SSU, LSU, and *cox 1* were aligned with published sequences for other acanthocephalans retrieved from the GenBank dataset. Alignments for each molecular marker (SSU, LSU, and *cox1*) were built using Clustal W (Thompson et al., 1994). A nucleotide substitution model was selected for the dataset using jModelTest version 2.1.7 (Posada, 2008). Phylogenetic analyses were inferred through maximum likelihood (ML) with RAxML version 7.0.4 (Stamatakis, 2006). A GTRGAMMAI substitution model was used, and 10,000 bootstrap replicates were run to assess nodal support. In addition, a Bayesian analysis was carried out, using MrBayes 3.2.2 (Ronquist et al., 2012), with two Markov chain Monte Carlo (MCMC) runs for 10 million generations, sampling every 1000 generations, a heating parameter value of 0.2 and a burn-in of 25%. The resulting phylogenetic trees were visualized and edited using FigTree version 1.4.2 (Rambaut & Drummond, 2007). Finally, uncorrected *p* distances were estimated using the MEGA program version 11 (Kumar et al., 2016).

## Results

Taxonomy

Class: Palaeacanthocephala Meyer, 1931

Order: Echinorhynchida Southwell & Macfine, 1925

Family: Rhadinorhynchidae Lühe, 1912

Genus: *Rhadinorhynchus* Lühe, 1911

Species: *Rhadinorhynchus trachinoti* **n. sp.**

Type host: Gafftopsail pompano (*Trachinotus rhodopus* Gill)

Site of infection: Small intestine (prevalence 32%, (28/87)).

Type locality: off the coast of Mazatlán, Mexico (23°12′ 37.15″ N, 106° 25′ 28.9″ W).

Intermediate host: *Metamysidopsis frankfiersi* Hendrickx & Hernández-Payán, 2023 (Crustacea: Mysidae).

Site of infection: Hemocoel (prevalence 3%, (50/1,613)

Locality: Playa Novillero, Nayarit, Mexico (22° 22′ 41.3″ N, 105° 41′ 11.4″ W).

Type-material: CNHE: 12139 (holotype); 12140 (allotype); 12141 (paratypes); 12142 (hologenophore)

Representative DNA sequences: PQ549640-641 (SSU); PQ549642-643 (LSU); PQ541022-025 (*cox 1*).

ZooBank registration (LSID): urn:lsid:zoobank.org:act:9BFA5D5A-A276-43B0-8A55-634D5DD25975

Etymology: The species name refers to genus of the host species in which the species was found along the Pacific coast of Mexico, *Trachinotus*.

Description

**Figure 3:**
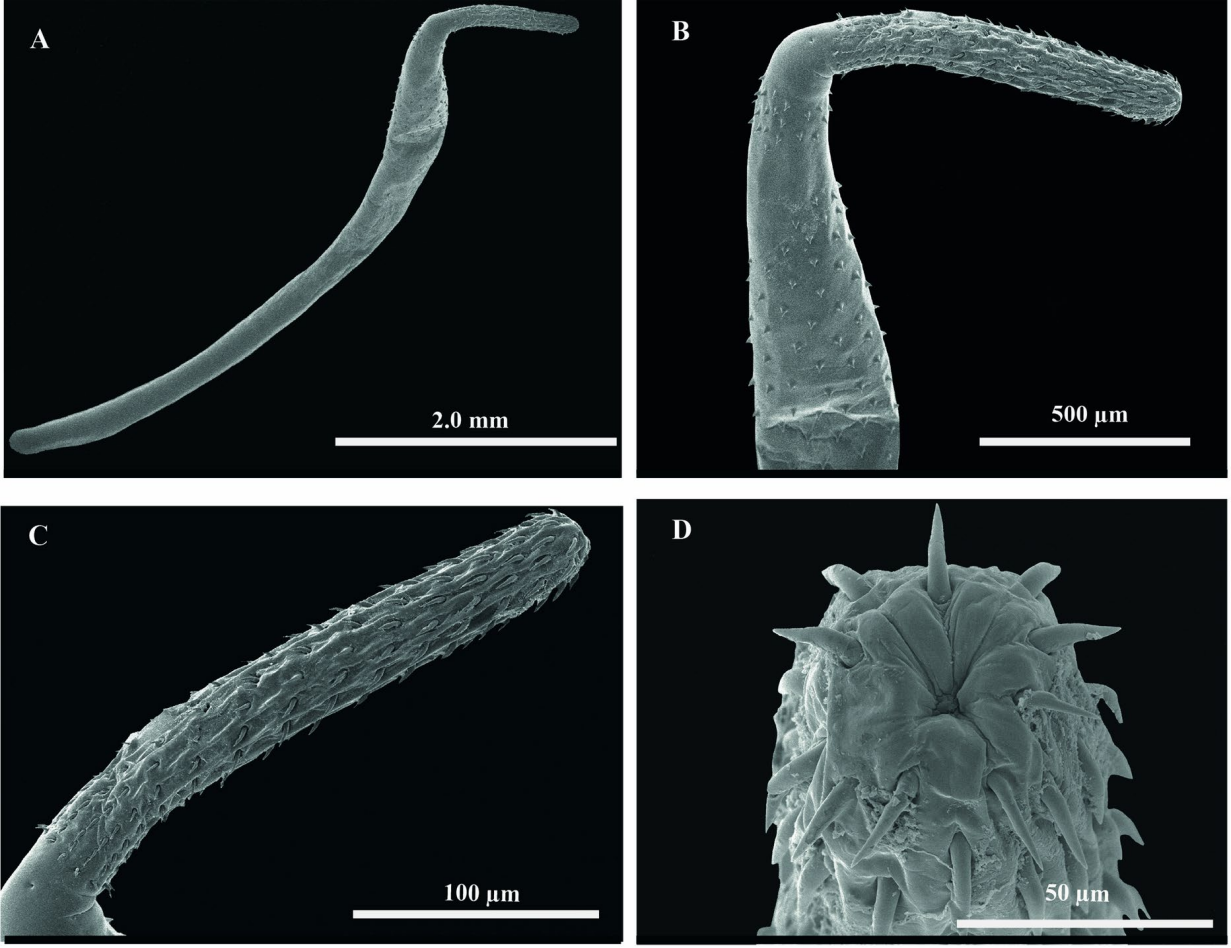
Scanning electron micrographs of ***Rhadinorhynchus trachinoti*** **n. sp.**, from *Trachinotus rhodopus* from off Mazatlán, Sinaloa, Mexico. Adult male, whole worm (**A**); Anterior region (**B**); Proboscis (**C, D**)

*Rhadinorhynchus trachinoti* **n. sp.** (Figs. 1–3)

General:

Sexual dimorphism evident, females larger than males. Trunk long uniformly cylindrical, covered with tegumental spines on the anterior region forming 2 fields separated by an unarmed zone (Fig. 1C, 3A–B). Anterior field with 3–4 irregular circle rows with 11–14 tegumental spines (Fig. 1C, 3B). Dorsal spines length 26–44; ventral spines length 24–40. Posterior field with 12–14 irregular circle rows with 13–14 tegumental spines. Dorsal spines length 30–45; ventral spines length 30–47, extending to level of 3/4 of proboscis receptacle length in both sexes. Proboscis long, cylindrical, armed with 12 longitudinal rows bearing 16–18 hooks each, decreasing in size towards posterior end except at basal ring where they become slightly larger (Fig. 1F, 3C–D). Neck smooth. Proboscis receptacle long, with cephalic ganglion near its middle (Fig. 1C). Lemnisci elongate, shorter than proboscis receptacle. Gonopore terminal in both sexes (Fig. 1C).

*Male* (based on 8 mounted specimens and 2 for SEM). Trunk, 5.9 mm long (5.2–6.6) × 490 (422–586) wide. Proboscis 1123 (1049–1188) × 167 (108–211), with 12 longitudinal rows with 16 hooks each, decreasing in size towards posterior end (Fig. 1D; Table 1). Neck 190 (164–258) × 180 (152–201). Proboscis receptacle 1945 (1755–2178) × 194 (158–225). Lemnisci almost of same size, 1213 (1100–1315) × 104 (89–121). Testes oblong contiguous, in posterior third of trunk (Fig. 1C). Anterior 563 (463–693) × 230 (167–304), posterior 552 (419–667) × 257 (211–288). Cement glands 4, in two pairs of different length, 1125 (842–1474) × 92 (67–133), 984 (669–1227) × 102 (80–133). Säeftingen’s pouch clavate 615 (402–740) × 162 (113–200) (Fig. 1C). Copulatory bursa partially-everted, 131 × 70 (n=1).

**Table 1:** Comparison between the size of dorsal and ventral proboscis hooks of 3 males and 3 females of ***Rhadinorhynchus trachinoti*** **n. sp.**

No. Hooks	Male		Female	
	Dorsal hooks	Ventral hooks	Dorsal hooks	Ventral hooks
1	47 (41–54)	51 (50–53)	44 (41–47)	44 (42–49)
2	56 (51–61)	49 (47–51)	49 (41–59)	52 (51–53)
3	55 (50–60)	53 (51–55)	56 (54–60)	49 (44–53)
4	54 (49–60)	49 (48–50)	57 (56–58)	53 (50–57)
5	54 (50–58)	52 (49–56)	55 (50–62)	54 (50–60)
6	54 (52–56)	53 (49–57)	53 (50–57)	52 (43–60)
7	49 (48–50)	56 (51–61)	56 (54–59)	53 (51–57)
8	54 (54)	54 (50–58)	52 (48–55)	52 (44–62)
9	52 (49–55)	53 (49–57)	50 (48–52)	55 (45–60)
10	48 (46–50)	43 (39–48)	57 (52–65)	54 (50–57)
11	35 (40–41)	32 (28–36)	54 (47–64)	50 (46–53)
12	32 (32)	29 (28–31)	38 (33–48)	40 (37–45)
13	36 (32–39)	28 (20–37)	35 (31–38)	32 (28–37)
14	30 (26–35)	23 (20–27)	30 (25–36)	33 (30–36)
15	29 (23–36)	24 (19–30)	23 (22–26)	29 (27–33)
16	40 (36–45)	40 (39–41)	23 (22–25)	26 (25–28)
17			23 (22–25)	24 (22–25)
18			37 (36–38)	36 (35–38)

*Female* (based on 10 mounted specimens). Trunk, 7.5 mm long (5.2–8.6) × 483 (395–527) wide. Proboscis 1208 (1166–1263) × 180 (112–210), with 12 longitudinal rows with 18 hooks each, decreasing in size towards the posterior end (see Table 1). Neck 186 (128–219) × 201 (181–231). Proboscis receptacle 2306 (1927–2583) × 192 (134–256). Lemnisci, 1464 (1347–1647). Reproductive system long, 2479 (1610–3198). Egg 32 (22–35) × 15 (9–16) (Fig. 1E, G).

*Cystacanth* (Fig. 2)

*Male* (based on 4 immature mounted specimens, and 1 for SEM). Trunk 4.2 mm long (3.9–4.5 mm) × 332 (285–400); maximum width at anterior region (Fig. 2B–C). Tegumental spines on the anterior region of trunk (Fig. 3E). Proboscis invaginated, (partially everted in SEM). (Fig. 2C–D). Proboscis receptacle 1.4 (1.4–1.6 mm) × 197 (183–207). Testes oblong contiguous in posterior third of trunk; anterior testis 299 (225–355) × 121 (101–159); posterior testis 302 (277–341) × 116 (82–134). Four tubular cement glands, 526 (450–612 mm) long (Fig. 2B).

*Female* (based on 1 immature mounted specimen). Trunk 4.5 mm long × 316, maximum width anterior region. Tegumental spines on the anterior region of trunk in 2 fields separates by aspinose zone. Proboscis invaginated. Proboscis receptacle 1272 long × 201 width. Reproductive system poorly developed.

*Remarks*

*Rhadinorhynchus trachinoti* **n. sp.** is morphologically characterized by having an elongated cylindrical trunk covered with conical tiny tegumental spines in the anterior region extended in two fields of irregular spine circles, elongated cylindrical proboscis armed with hooks of different size and shape, a cephalic ganglion located at the middle of proboscis receptacle, lemnisci shorter than receptacle proboscis, four cement glands, and gonopore terminal. This combination of characters allocates this species into the genus *Rhadinorhynchus* (Amin et al., 2011; Smales, 2014; Lisitsyna et al., 2019). The morphological traits exhibited by *Rhadinorhynchus trachinoti* **n. sp.**, are unique and they are not shared with other congeneric species; the new species represent the eighth species described in marine fishes of the Americas. *Rhadinorhynchus trachinoti* **n. sp.** can be differentiated morphologically from other seven congeneric species reported from marine fishes of the Americas by having a proboscis with the smallest number of hooks per row, since it is armed with 12 longitudinal rows bearing 16–18 hooks each (Figs. 1D; 3B–D), whereas the proboscis of *R. cololabis* Larus & McCauley, 1964 is armed with 10–12 longitudinal rows with 20–21 hooks each; *R. dujardini* Golvan, 1969, with 22–25 longitudinal rows with 30–40 hooks each; *R. ornatus* Van Cleave, 1918, with 22–24 longitudinal rows with 38–40 hooks each; *Rhadinorhynchus decapteri* (Braicovich, Lanfranchi, Farber, Marvaldi, Luque & Timi, 2014) Huston, Cribb & Smales, 2020, with 10 longitudinal rows with 22–26 hooks each; *Rhadinorhynchus oligospinosus* Amin & Heckmann, 2017, with 11–14 longitudinal rows but possessing 20–22 hooks each; *Rhadinorhynchus selkirki* Van Cleave, 1921 with 12 longitudinal rows as in the new species but with 24 hooks each; and *R. trachuri* Harada, 1935, with 12 longitudinal rows with 19–24 hooks each which probably is one of the most widely distributed species since it is found in the Sea of Japan, the Indian Ocean, and the Pacific coast of South and North America, in association with salmonids and carangid fishes (see Van Cleave, 1921; Amin et al., 2011, 2019; Amin, 2020; Braicovich et al., 2014; Amin & Heckmann, 2017; Gibson & Wayland, 2024).

*Phylogenetic analyses*

**Figure 4:**
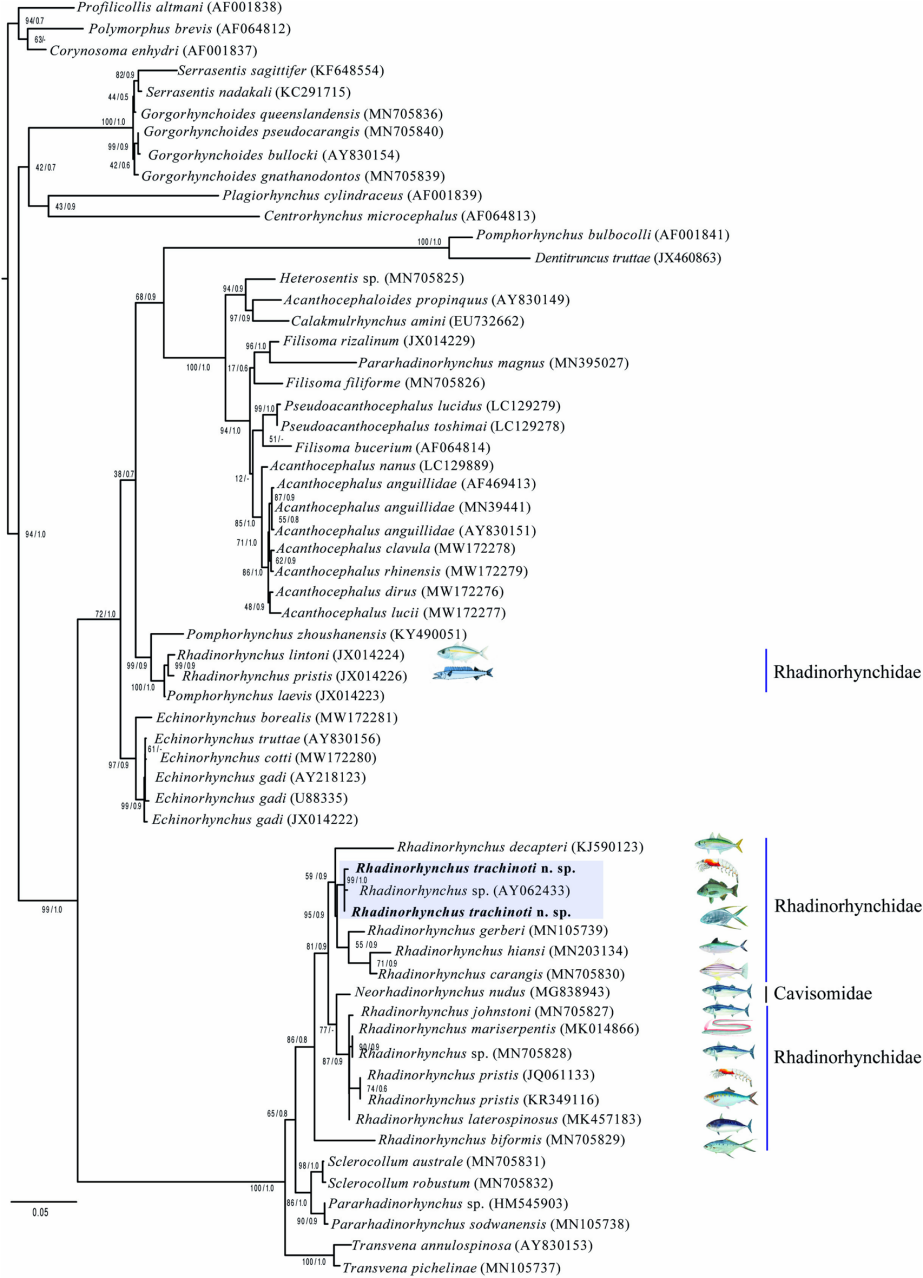
Phylogenetic trees using maximum likelihood (ML) and consensus Bayesian obtained with the small subunits of ribosomal DNA dataset. Numbers near internal nodes show ML bootstrap percentages/ Bayesian posterior probabilities

The alignment of the SSU consisted of 61 taxa and 1,844 bp, whereas the LSU alignment included 45 taxa with 3,077 bp. The phylogenetic trees inferred with the SSU dataset exhibited low phylogenetic signal, and recovered *Rhadinorhynchus* as paraphyletic, due that two subclades were formed. The first subclade contained *R. lintoni* Cable & Linderoth, 1963 (JX014224) and *R. pristis* Rudolphi, 1802 (JX014226) as the sister group of species of *Pomphorhynchus* (Zoega, 1776), a relationship supported with high bootstrap (99%) and Bayesian posterior probability support values (0.9) (Fig. 4). The second subclade contained 11 species of *Rhadinorhynchus*, including the new species, plus *Neorhadinorhynchus nudus* (Harada, 1938) Yamaguti, 1939 (MG838943). Within the subclade, the two sequences of *Rhadinorhynchus trachinoti* **n. sp.**, obtained from its definitive and intermediate were nested with a sequence of *Rhadinorhynchus* sp. (AY062433) from a marine fish off the coast of Chamela Bay, in the western Pacific coast of Mexico, and were retrieved as a sister a clade of *R. gerberi* Lisitsyna, Kudlai, Cribb and Smit, 2019 (MN105739), and *R. carangis* Yamaguti, 1939 (MN705830) plus *R. hiansi* Soota & Bhattacharya, 1981(MN203134), with the high bootstrap (95%) and Bayesian posterior probability support values (0.9) (Fig. 4).

**Figure 5:**
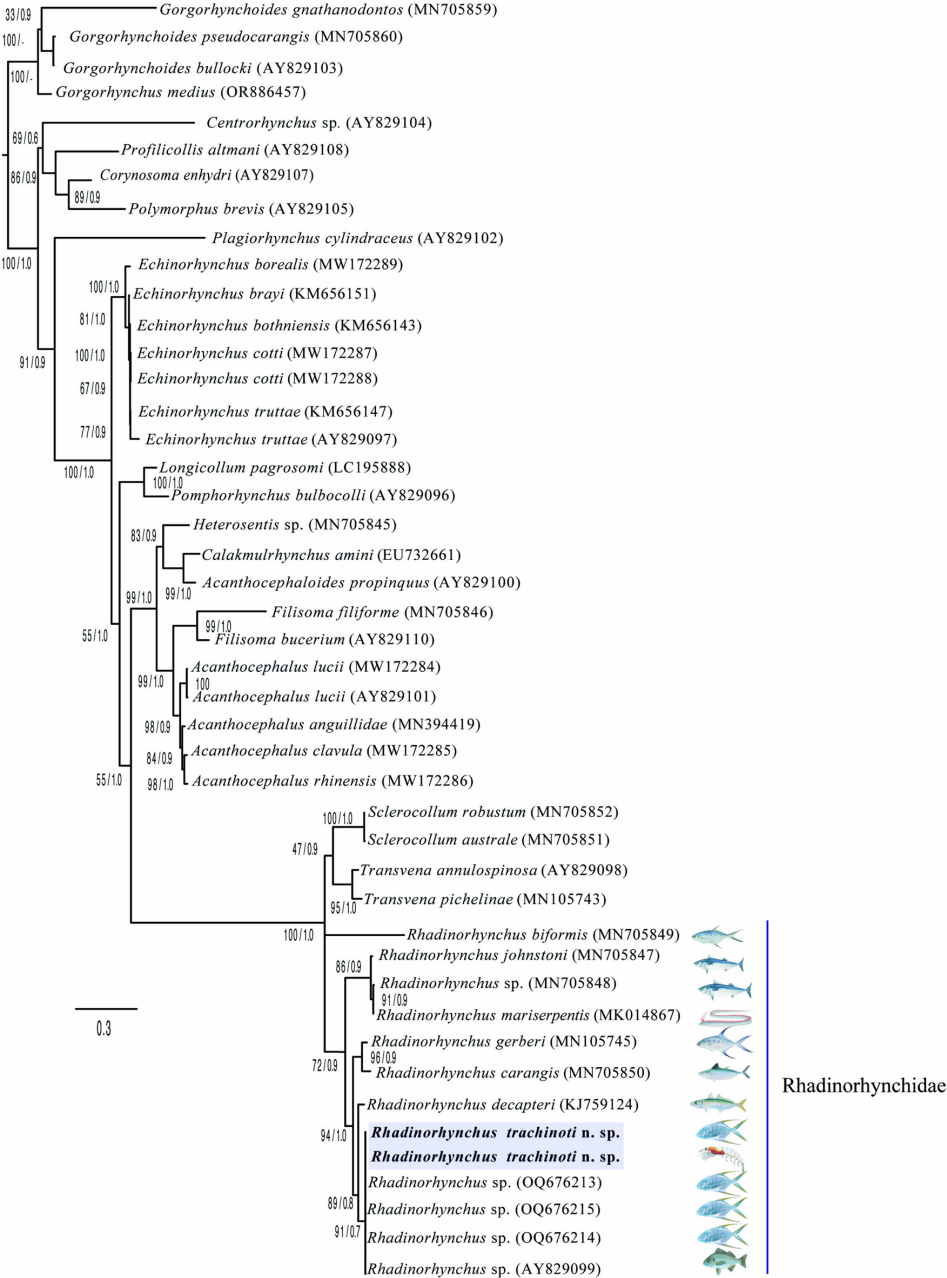
Phylogenetic trees using maximum likelihood (ML) and consensus Bayesian obtained with the large subunits of ribosomal DNA dataset. Numbers near internal nodes show ML bootstrap percentages/ Bayesian posterior probabilities

The phylogenetic trees inferred with the LSU dataset yielded a different topology. Taxa representation for both molecular markers is somewhat different, although both analyses agreed on the systematic position of the taxa under study. The genus is retrieved forming a polytomy along with species of *Transvena* Pichelin & Cribb, 2001 and *Sclerocollum* Schmidt & Paperna, 1978. However, the new sequences of *Rhadinorhynchus trachinoti* **n. sp.**, obtained from its definitive and intermediate hosts plus four isolates identified as *Rhadinorhynchus* sp., (AY829099, OQ676213–215) from the Gafftopsail pompano (*T. rhodopus*) from two localities of the Pacific coast of Mexico (Chamela Bay, Jalisco, and Puerto Angel Bay, Oaxaca, respectively) formed a subclade with high bootstrap (91%) and Bayesian posterior probability support values (0.7) (Fig. 5). The new species was retrieved as the sister group of *R. decapteri* (KJ759124), which was described from a carangid from the coastal waters of Brazil in the western Atlantic. These two species were yielded as sister taxa of *R. carangis* Yamaguti, 1939 (MN705850) plus *R. gerberi* (MN105745) (Fig. 5).

**Figure 6:**
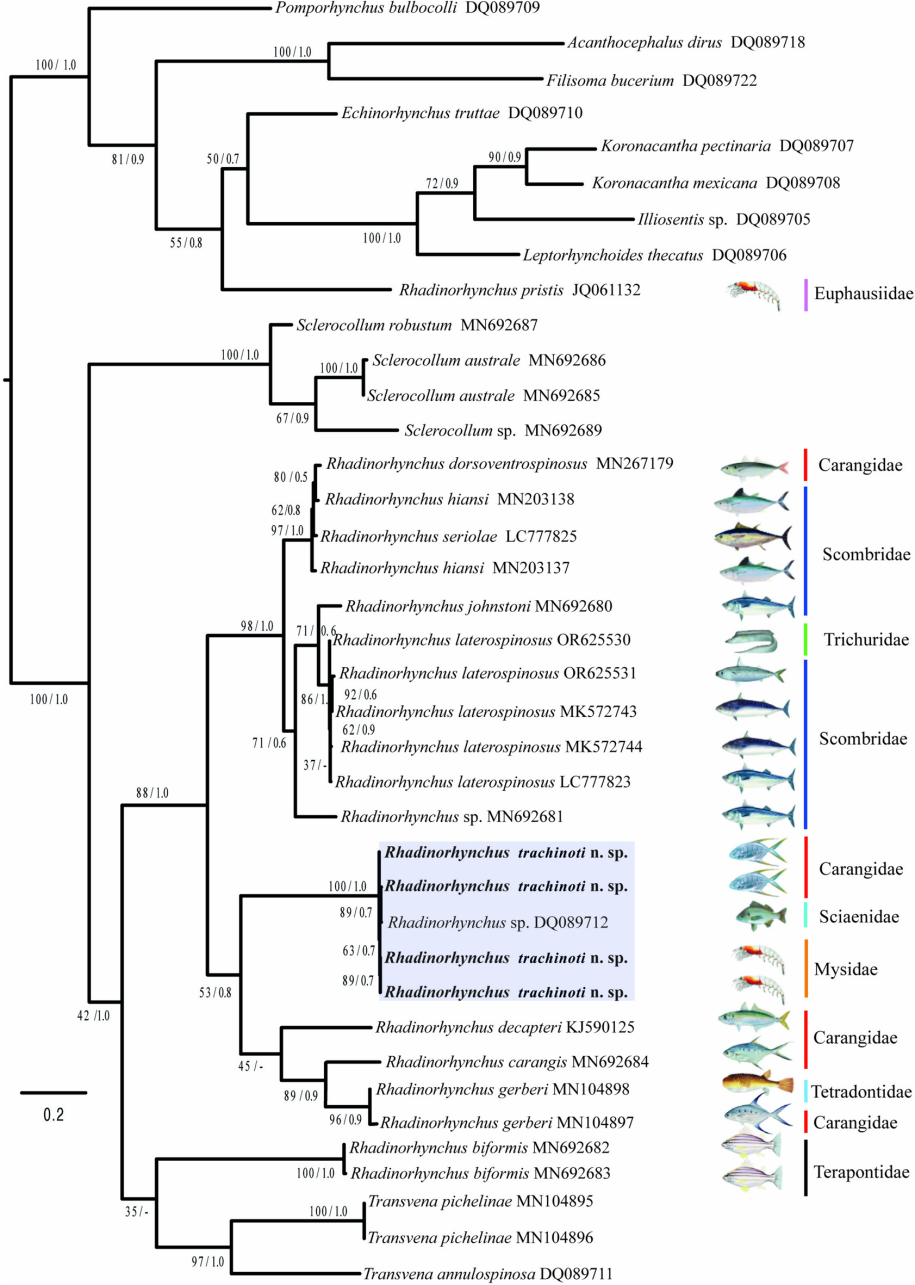
Phylogenetic trees using maximum likelihood (ML) and consensus Bayesian obtained with the cytochrome c oxidase subunit 1 from the mitochondrial DNA dataset. Numbers near internal nodes show ML bootstrap percentage values/ Bayesian posterior probabilities

The *cox 1* alignment included 655 bp and 38 sequences. The phylogenies inferred with the ML and BI methods also yielded *Rhadinorhynchus* as paraphyletic, since three species of *Transvena* nested as the sister group of two isolates of *R. biformis* (MN692682–683), albeit with very low bootstrap and Bayesian posterior probabilities support values (Fig. 6). All other species of *Rhadinorhynchus* formed a clade containing eight species of which two isolates of *R. gerberi* (MN104897–898)*, R. carangis* (MN692684), and *R. decapteri* (KJ590125) appeared as sister the sister group of four isolates of *Rhadinorhynchus trachinoti* **n. sp.**, a similar result as in the 28S phylogenetic tree. Furthermore, the sequences of the new species obtained from its definitive and intermediate hosts nested in a clade along with an isolate identified as *Rhadinorhynchus* sp., (DQ089712) from off Chamela Bay, Mexico. The sister clade contained a group of six species plus an unidentified species of *Rhadinorhynchus* (MN692681), including *R. laterospinosus* Amin, Heckmann, Ha, 2011 (OR625530–531, MK572743–744, LC777823), *R. johnstoni* (MN692680), *R. hiansi* (MN203137–138), *R. seriolae* (Yamaguti, 1963) (LC777825) and *R. dorsoventrospinosus* Amin, Heckmann, Ha, 2011 (MN267179) (Fig. 6). The genetic divergence estimated among the four isolates of *Rhadinorhynchus trachinoti* **n. sp.**, plus an unidentified isolate of *Rhadinorhynchus* sp., ranged from 0.0% to 0.06%., whereas the genetic divergence among the new species and *R. decapteri*, *R. gerberi*, and *R. carangis*, their sister taxa, varied from 24 to 26%.

## Discussion

Adults of the genus *Rhadinorhynchus* are known to infect the intestines of marine fishes of the families Carangidae, Salmonidae, Scombridae Terapontidae, Tetradontidae, and Trichuridae worldwide (Amin, 2020). The new species, *Rhadinorhynchus trachinoti* **n. sp.**, is herein recorded from the intestine of the Gafftopsail pompano (*T. rhodopus*), a carangid distributed mostly in the Eastern Tropical Pacific (Thomson et al., 1979), representing the eighth species of *Rhadinorhynchus* in the Americas, and the first species along the Pacific coast of Mexico. The new species is distinguished from other seven congeneric species from the Americas by having a proboscis cylindrical, armed with 12 longitudinal rows with 16–18 hooks each. The molecular data obtained from the adult specimens of *Rhadinorhynchus trachinoti* **n. sp.**, were key to link them with sequences obtained from the cystacanths recovered from the mysid (*M. frankfiersi*) from a nearby locality. The phylogenetic analyses performed with the SSU and LSU from nuclear ribosomal DNA, placed the two isolates (one adult and one cystacanth) in a single clade, suggesting that the two isolates are conspecific. The current evidence suggested that the isolates of *Rhadinorhynchus* sp., from the Chamela Bay, Jalisco, on the Pacific coast of Mexico available in GenBank (AY062433; SSU and AY829099; LSU), and OQ676213–215 from Puerto Angel, Oaxaca, also from the Mexican Pacific coast, should be considered as *Rhadinorhynchus trachinoti* **n. sp.**, because the isolates were nested in the same clade together with the sequences of the new species we describe herein. In addition, sequences of the *cox 1* from the mitochondrial DNA were generated and analyzed and confirmed that the adult specimens and the cystacanths were conspecific. The phylogenetic analyses inferred with *cox1*, placed all isolates (two adults and two cystacanths) of *Rhadinorhynchus trachinoti* **n. sp.**, together with an isolate identified as *Rhadinorhynchus* sp., available in GenBank (DQ089712) from Chamela Bay in a single clade. The intraspecific genetic divergence among five isolates was very low, ranging from 0 to 0.06%. The level of intraspecific genetic variation found is similar to that found in other species of *Rhadinorhynchus*. For example, the genetic divergence between two isolates of *R. gerberi* which parasitizes the Largespotted dart (*Trachionotus botla* Lacépède) in South Africa was 0.4% (Lisitsyna et al., 2019); whereas the divergence between two isolates of *R. biformis* from the Eastern striped trumpeter (*Pelates sexlineatus* Quoy & Gaimard) from Queensland, Australia was also 0.4% (Huston et al., 2020). Lisitsyna et al. (2023) also compared three isolates of *R. laterospinosus* from the Largehead hairtail (*Trichiurus lepturus* Linnaeus) and the Blue mackerel (*Scomber australasicus* Cuvier) from the East China Sea, and the Bullet tuna (*Auxis rochei* Risso) from the South China Sea, and the divergence was 0.05%.

In our study, adults of *R. trachinoti* **n. sp.** were found parasitizing the intestine of the Gafftopsail pompano (*T. rhodopus*). The helminth fauna of this host species is relatively well known throughout its distribution range in Mexican coasts, and a large diversity of parasites has been documented (Martínez-Flores et al., 2023; Osuna-Cabanillas et al., 2024). One of the parasites reported in this host species is the acanthocephalan which was not identified until the present study, as a new species, and because of that *R. trachinoti* **n. sp.** could be considered to be a typical component of the helminth fauna of this fish species. Furthermore, the cystacanths of the species in the crustacean intermediate hosts show a low prevalence value; of the 1,613 mysids examined only 3% were infected; we report a mysid crustacean for the first time as the intermediate host of species of *Rhadinorhynchus*. In a previous study, the euhausiid *Nyctiphanes couchii* Bell was reported as the intermediate host of *Rhadinorhynchus* sp., in NW Iberian Peninsula waters (Gregori et al., 2013). Although the complete life cycles of the species of *Rhadinorhynchus* are unknown, the current evidence, suggest that adult worms of the genus *Rhadinorhynchus* live and reproduce sexually in the digestive tracts of fish. Female worms release eggs that are expelled into the environment with the faeces of the host. After ingestion of the eggs by a mysid or an euphausiid that serve as the intermediate hosts, the parasite develops into a juvenile or cystacanth stage, and the parasite develops into the adult stage within the digestive tract of marine fishes.

The data obtained in this study suggest a distribution pattern for *R. trachinoti* **n. sp.** along the Pacific shoreline of Mexico, extending from Sinaloa in the north to the coast of Oaxaca in the south. This pattern aligns with the marine current that flows from the Gulf of Tehuantepec to the entrance of the Gulf of California, serving as a bridge between tropical waters and subtropical coastal regions in the northeastern Pacific (Zamudio et al., 2007; Gómez-Valdivia et al., 2015).

Finally, our study represents a step forward in the understanding of the transmission and life cycle of *Rhadinorhynchus* species. The current evidence indicated that the malacostracans which include mysid and euphausiids serve as intermediate hosts to species of *Rhadinorhynchus* and there is no evidence at the moment that the infection with the cystacanths produce an alteration on the behavior or coloration of their intermediate hosts; this needs to be corroborated through further samplings. The fact that crustaceans infected with cystacanths generate a substantial shift in behavior and coloration, preferring areas of high light intensity facilitating the depredation by its definitive hosts is a well-known phenomenon (Kennedy, 2006).

## Data Availability

No datasets were generated or analysed during the current study.

## References

[CR1] Amin, O. M. (2020). Redescription of *Rhadinorhynchus trachuri* Harada, 1935 (Acanthocephala: Rhadinorhynchidae) from Marine Fish in Vietnam and California with a discussion of its zoogeography. *Acta Parasitologica*, 65, 77–89. 10.2478/s11686-019-00130-z. 31586284 10.2478/s11686-019-00130-z

[CR2] Amin, O. M., & Heckmann, R. A. (2017). *Rhadinorhynchus oligospinosus* n. sp. (Acanthocephala, Rhadinorhynchidae) from mackerels in the Pacific Ocean off Peru and related rhadinorhynchids in the Pacific, with notes on metal analysis. *Parasite,* 24: 19. 28593837 10.1051/parasite/2017022 PMC5467225

[CR3] Amin, O. M., Heckmann, R., A., & Ha, N. V. (2011). Description of two new species of *Rhadinorhynchus* (Acanthocephala, Rhadinorhynchidae) from marine fish in Halong Bay, Vietnam with a key to species. *Acta Parasitologica,* 56 (1), 67–77. 10.2478/s11686-011-0004-3.

[CR4] Amin, O. M., Rubtsova, N, Y., & Ha, N. V. (2019). Description of three new 31270659 10.2478/s11686-019-00092-2

[CR7] Andrade-Gómez, L., & García-Varela, M. (2021). Unexpected morphological and molecular diversity of trematode (Haploporidae: Forticulcitinae) parasites of mullets from the ocean Pacific coasts in Middle America. *Parasitology Research*, 120 (1), 55–72. 33247332 10.1007/s00436-020-06983-y

[CR9] Braicovich, P. E., Lanfranchi, A. L., Farber, M. D., Marvaldi, A. E., Luque, J. L. & Timi, J. T. (2014). Genetic and morphological evidence reveals the existence of a new family, genus and species of Echinorhynchida (Acanthocephala). *Folia Parasitologica*, 61(4), 377–84. 10.14411/fp.2014.044 25185409

[CR10] Danemann, G. D. (1993). Características generales de la dieta de la palometa (*Trachinotus rhodopus* (Perciformes: Carangidae). *Revista de Biología Tropical,* 41(3), 811–815.

[CR11] Folmer, O., Black, M., Hoeh, W., Lutz, R., & Vrijenhoek, R. (1994). DNA primers for the amplification of mitochondrial cytochrome c oxidase subunit I from diverse metazoan invertebrates. *Molecular Marine Biology and Biotechnology*, 3(5), 294–299. 7881515

[CR12] García-Prieto, L., García-Varela, M., Mendoza-Garfias, B., & Pérez-Ponce de León, G. (2010). Checklist of the Acanthocephala in wildlife vertebrates of Mexico. *Zootaxa,* 2419, 1–50. 10.11646/zootaxa.2419.1.1

[CR13] García-Varela, M., & Nadler, S. A. (2005). Phylogenetic relationships of Palaeacanthocephala (Acanthocephala) inferred from SSU and LSU rDNA gene sequences. *Journal of Parasitology*, 91(6), 1401–1409. 10.1645/GE-523R.1 16539024 10.1645/GE-523R.1

[CR14] García-Varela, M., Sereno-Uribe, A. L., Solórzano-García, B., & Pérez-Ponce de León, G. (2024). The white grunt, *Haemulon plumierii* (Lacepède, 1801) as paratenic and definitive host of two acanthocephalan species, with the description of a new species of *Dollfusentis* (Palaeacanthocephala: Leptohynchoididae) from the Yucatán Peninsula, Mexico. 38584424 10.1017/S0022149X24000105

[CR16] Gibson, D. & Wayland, M. (2024). World List of marine Acanthocephala. *Rhadinorhynchus* Lühe, 1911. Accessed through: World Register of Marine Species at: https://www.marinespecies.org/aphia.php?p=taxdetails&id=20399 on 2024-09-25

[CR17] Gómez-Valdivia, F., Parés-Sierra, A., & Flores-Morales, A. L. (2015). The Mexican coastal

[CR20] Gregori, M., Aznara, F. J., Abollo, E., Roura, A., González, A. F., & Pascual, S. (2013).

[CR23] Hendrickx, M. E., & Hernández-Payán, J. C. (2023). The genus *Metamysidopsis* W.M. Tattersall, 1951 (Peracarida, Mysida, Mysidae) in the eastern Pacific with the description of a new species from western Mexico and notes on some diagnostic characters used in the genus. *Crustaceana*, 96, 423‒453. 10.1163/15685403-bja10293

[CR24] Huston, D, C., Cribb, T. H., & Smales, L. R. (2020). Molecular characterisation of acanthocephalans from Australian marine teleosts: proposal of a new family, synonymy of other and transfer of taxa between orders. *Systematic Parasitology,* 97, 1–23. 10.1007/s11230-019-09896-2 31912420 10.1007/s11230-019-09896-2

[CR26] Kennedy, C. R. (2006). Ecology of the Acanthocephala. Cambridge University Press. New York.

[CR27] Kumar, S., Stecher, G., & Tamura, K. (2016). MEGA7: Molecular Evolutionary Genetics Analysis version 7.0 for bigger datasets. *Molecular Biology and Evolution*, 33(7), 1870–1874, 10.1093/molbev/msw054. 27004904 10.1093/molbev/msw054 PMC8210823

[CR28] Lisitsyna, O. I., Kudlai, O., Cribb, T. H., & Smit N. J. (2019). Three new species of acanthocephalans (Palaeacanthocephala) from marine fishes collected off the East coast of South Africa. *Folia Parasitologica*, 66, 012, 10.14411/fp.2019.012. 10.14411/fp.2019.012 31558687

[CR29] Lisitsyna, O. I., Barčák, D., Orosová, M., Fan, C. K., & Oros, M. (2023). Acanthocephalans of marine and freshwater fishes from Taiwan with description of a new species. *Folia Parasitologica*, 70, 021. 10.14411/fp.2023.021. 10.14411/fp.2023.021 38167244

[CR30] Martínez-Flores, G., García-Prieto, L., Bastida-Zavala., & Oceguera-Figueroa, A. (2023). Temporal variation in helminth infracommunities of the Gafftopsail pompano, *Trachinotus rhodopus* (Pisces: Carangidae) off the Pacific coast of Mexico. *Parasitology International,* 95, 102755. 10.1016/j.parint.2023.102755 37137347 10.1016/j.parint.2023.102755

[CR31] Osuna-Cabanillas, J M., Marín-Enríquez, E., Martínez-Falcón A. P., Timi J. T., & Morales-Serna F. N. (2024). Low similarity between parasite communities of ten sympatric carangid species. *Parasitology International,* 101, 102885. 10.1016/j.parint.2024.102885. 38461933 10.1016/j.parint.2024.102885

[CR32] Pérez‐Ponce De León, G., García‐Prieto, L., & Mendoza-Garfías, B. (2007). Trematode parasites (Platyhelminthes) of wildlife vertebrates in Mexico. *Zootaxa*, 1534(1), 1–247. 10.11646/zootaxa.1534.1.1.

[CR33] Pleijel, F., Jondelius, U., Norlinder, E., Nygren, A., Oxelman, B., Schander, C., Sundberg, P., & Thollesson, M. (2008). Phylogenies without roots? A plea for the use of vouchers in molecular phylogenetic studies. *Molecular Phylogenetics and Evolution*, 48(1), 369–71. 10.1016/j.ympev.2008.03.024. 18424089 10.1016/j.ympev.2008.03.024

[CR34] Posada, D. (2008). jModelTest: phylogenetic model averaging. *Molecular Biology and Evolution*, 25(7), 1253–1256. 10.1093/molbev/msn083 18397919 10.1093/molbev/msn083

[CR35] Rambaut, A., & Drummond, A. J. (2007). Tracer v1.4, http://beast.bio.ed.ac.uk/Tracer 2007 accessed.

[CR36] Ronquist, F., Teslenko, M., Van Der Mark, P., Ayres, D. L., Darling, A., Höhna, S., Larget, B., Liu, L., Suchard, M. A., & Huelsenbeck, J. P. (2012). MrBayes 3.2: Efficient Bayesian phylogenetic inference and model choice across a large model space. *Systematic Biology*, 61(3), 539–542. 10.1093/sysbio/sys029 22357727 10.1093/sysbio/sys029 PMC3329765

[CR37] Santos-Bustos, N. G., Violante-González J., Monks, S., Rojas-Herrera, A. A., Flores- Rodríguez, P., Rosas-Acevedo, J. L., & Villalba-Vasquez, P. J. (2021). Parasite communities of striped bonito *Sarda orientalis* (Pisces: Scombridae) on the Pacific coast of Mexico. *New Zealand Journal of Zoology,* 48, 97–112. 10.1080/03014223.2020.1792516.

[CR38] Smales L. R. (2014). The genus *Rhadinorhynchus* (Acanthocephala: Rhadinorhynchidae) from marine fish in Australia with the description of four new species. *Acta Parasitologica,* 59, 721–736. 10.2478/s11686-014-0305-4 25236286 10.2478/s11686-014-0305-4

[CR39] Stamatakis, A. (2006). RAxML-VI-HPC: maximum likelihood-based phylogenetic analyses with thousands of taxa and mixed models. *Bioinformatics*, 22(21), 2688–2690. 16928733 10.1093/bioinformatics/btl446

[CR41] Thompson, D. A., Findley, L. T., & Kerstitch, A. N. (1979). Reef fishes of the Sea of Cortez. The rocky-shore fishes of the Gulf of California. University of Arizona Press, Tucson, Arizona, E.U.A 302 p.

[CR42] Thompson, J. D, Higgins, D. G., & Gibson, T. J. (1994). CLUSTAL W: improving the sensitivity of progressive multiple sequence alignment through sequence weighting, position-specific gap penalties and weight matrix choice. *Nucleic Acids Research*, 22(22), 4673–4680. 10.1093/nar/22.22.4673. 7984417 10.1093/nar/22.22.4673 PMC308517

[CR43] Van Cleave, H. J. (1921). Acanthocephala collected by the Swedish Expedition to the Juan Fernandez Islands (1916-1917). *The Natural History of Juan Fernandez and Easter Island,* 3, 75.

[CR44] Van Cleave, J. H. (1940). The Acanthocephala collected by the Allan Hancock Pacific Expedition, 1934. *University of South California Publications*, 2, 501–516.

[CR45] Villalba-Vasquez, P.J., Violante-González, J., Pulido-Flores, G., Monks, S., Rojas- Herrera, A. A., Flores-Rodríguez, P., Rosas-Acevedo, J. L., Valencia-Cayetano, C., & Santos-Bustos, N. G. (2022). Parasite communities of the spotted rose snapper *Lutjanus guttatus* (Perciformes: Lutjanidae) off the Mexican Pacific coasts: spatial and long-term inter-annual variations. *Parasitology International,* 8, 102551. 10.1016/j.parint.2022.102551. 10.1016/j.parint.2022.102551 35101604

[CR46] Violante-González, J., Gallegos-Navarro, Y., Monks, S., García-Ibáñez, S., Rojas- Herrera, A. A., Pulido-Flores, G., & Larumbe-Morán, E (2016). Parasites of the green jack *Caranx caballus* (Pisces: Carangidae) in three locations from Pacific coasts of Mexico, and their utility as biological tags. *Revista Mexicana de Biodiversidad*, 87,(3) 1015–1022. 10.1016/j.rmb.2016.07.010.

[CR47] Violante-González, J., Monks, S., Gallegos-Navarro, Y., Santos-Bustos, N. G., Villalba-Vásquez, P. J., Padilla-Serrano, J. G., & Pulido-Flores, G. (2020). Interannual variation in the metazoan parasite communities of bigeye trevally *Caranx sexfasciatus* (Pisces, Carangidae), *Parasite*, 27, 1–11. 10.1051/parasite/2020001. 32003324 10.1051/parasite/2020001 PMC6993563

[CR48] Zamudio, L., Hurlburt, H. E., Metzger, E. J., & Tilburg, C. E. (2007). Tropical wave induced oceanic eddies at Cabo Corrientes and the María Islands, Mexico. *Journal of Geophysical Research,* 1–17. 10.1029/2006JC004018.

